# Growth of community‐based immunotherapy treatment in the Veterans Health Administration

**DOI:** 10.1002/cam4.6372

**Published:** 2023-07-31

**Authors:** Megan Ellis Price, Sarah Gordon, Caroline Emmitt, Nambi Ndugga, Aigerim Kabdiyeva, Hillary Mull, Steven Pizer, Melissa M. Garrido

**Affiliations:** ^1^ VA Boston Medical Center Boston Massachusetts USA; ^2^ Boston University School of Public Health Boston Massachusetts USA; ^3^ Boston University School of Medicine Boston Massachusetts USA

**Keywords:** cancer management, clinical management, clinical guidelines, risk assessment, statistical methods

## Abstract

**Background:**

The MISSION and CHOICE Acts expanded the Veterans Health Administration's (VA) capacity to purchase immunotherapy services for VA patients from community‐based providers. Our objective was to identify predictors of community‐based immunotherapy treatment, and assess differences in cost and utilization across community treatment settings

**METHODS:**

We examined claims for 21,257 patients who started immunotherapy treatment between 2015 and 2020. We assessed growth in VA community‐based immunotherapy care, predictors of community‐based immunotherapy treatment using multivariable logistic regression based on patients' sociodemographic and clinical characteristics. We compared utilization and costs among those who received community‐based immunotherapy services in hospital outpatient departments (HOPDs) versus physician office settings (POs).

**Results:**

The proportion of community‐based immunotherapy in the VA increased from 5.3% in 2015 to 32.1% in 2020, with total annual costs of immunotherapy growing from $6.1 million to $187 million. Older, married, and rural patients and those with more comorbidities were more likely than younger, single, or urban patients to be treated in the community. Black patients were more likely to be treated in the VA. Respiratory Cancer was the most common cancer type in both settings. Among community immunotherapy patients, we observed no meaningful differences in the number of units administered, the unit drug costs, or the cost per immunotherapy visit between POs and HOPDs.

**Conclusion:**

Drug costs did not differ widely across HOPDs and POs among VA patients who receive community‐based immunotherapy.

## Graphical abstract

VA patients who received community‐based immunotherapy were more likely to live farther from a VA facility, reside in a rural area, or live near a lower complexity VA facility with lower chemotherapy volume access. Drug costs for Nivolumab and Pembrolizumab were similar across different settings of care.

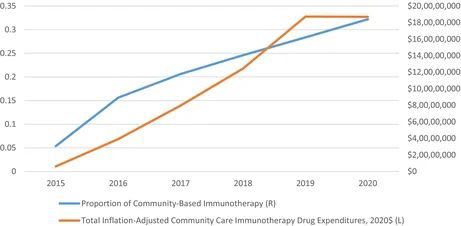

## 1 INTRODUCTION

Cancer is the second leading cause of death in the United States behind heart disease. The Veterans Health Administration (VA) is a high‐volume provider of cancer care. Nearly 50,000 incident cancer cases are identified within the VA annually and 3% of all cancer patients in the United States each year receive at least some portion of their care in the VA system.^1^, ^2^ Oncologic care is a significant cost burden for the VA, driven in part by novel treatments. In 2021, total spending on oncology drugs in the United States was $75 billion.^3^ Average growth in oncology drug costs was 10.5% a year from 2017 to 2021.^3^ The average drug costs for Nivolumab and Pembrolizumab, two commonly used immunotherapy treatments, are estimated to be approximately $75,000 and $55,000 per patient, respectively.^4^

In the VA, the high cost of immunotherapy care is compounded by the expansion of community‐based purchased care (“Community Care”). In 2014, the Choice Act enabled Veterans to see community‐based specialists if they lived further than 40 miles from a VA facility with a full‐time primary care provider, encountered wait times that exceeded a “reasonable period” (generally considered 30 days), or experienced hardship in obtaining care. The VA pays community‐based providers Medicare rates for seeing VA patients. In 2019, the MISSION Act further expanded the Community Care program by enabling Veterans to see a specialist if their drive time to the nearest VA facility exceeded 60 min or if they encountered wait times that exceeded 28 days. To date, the proportion and cost of community‐based immunotherapy within the VA has not been examined.

One potential cost reduction lever may be care setting, if costs differ between categories of care settings but do not affect quality of care. The site of cancer care has increasingly shifted from private physician offices to hospital‐affiliated outpatient infusion centers. Between 2012 and 2013, the number of hospital outpatient departments (HOPDs) increased by 25%.^5^ Among patients with Medicare and commercial insurance, higher costs for cancer care have been observed in HOPDs compared to physician offices (POs).^6^ One study found that Medicare fee‐for‐service costs for chemotherapy infusions were 14% higher in HOPDs compared to POs, and another study found that among commercially insured patients, the average cost per chemotherapy episode was 34% higher in HOPDs compared to POs.^7^, ^8^

The primary objective of this study was to quantify use of community‐based immunotherapy within the VA. The secondary objective was to compare costs between HOPDs and POs among VA patients receiving care in the community, to explore whether oncologic care setting could be a potential avenue for controlling immunotherapy costs within the VA. We used VA and community care claims and the Pharmacy IV database from 2015 to 2020 to (1) assess the proportion of immunotherapy treatment provided within the community versus the VA; (2) identify characteristics of patients receiving community‐based immunotherapy; and (3) to measure cost and utilization differences in community‐based immunotherapy care in HOPDs versus POs.

## 2 METHODS

### 2.1 Data and sample

We used data from the VA Corporate Data Warehouse from CY 2015 to 2020, including VA and community care claims. The community care claims include the PIT and Fee databases.^3^, ^9^, ^10^ We identified immunotherapy treatment based on CPT codes and text matching in community care claims data and based on text matching in VA pharmacy data (Appendix A). For the comparison between treatment at the VA and in the community, we selected five immunotherapy drugs, Nivolumab, Pembrolizumab, Atezolizumab, Sipuleucel‐T, and Ipilimumab, which were the first five FDA‐approved immunotherapy drugs that are applicable to a broad array of cancers.^11^ We had an initial sample of 24,123 patients. We removed 2866 patients who were missing location information or Elixhauser comorbidity scores (Appendix B). The final sample included claims for 21,257 VA patients, 15,452 patients who began immunotherapy treatment within the VA, and 5805 patients who started immunotherapy treatment in the community.

For the comparison of outpatient POs versus hospital outpatient departments, we limited the sample to 6108 patients who received care in the community and who received Nivolumab or Pembrolizumab, the two most commonly administered immunotherapy drugs at the VA. We also excluded 51 patients who were missing demographic information, and 279 additional patients who were treated in multiple settings or with multiple drugs. Finally, we trimmed outlier cost observations that we believe were data entry errors (four patients with average unit costs less than $1 and five patients with an average unit cost greater than $1000) (Appendix C).

This project was intended to inform VA operational practices and was deemed non‐research by the VA Boston IRB.

### 2.2 Measures

The primary outcome was the proportion of community‐based immunotherapy treatment defined as VA‐financed immunotherapy delivered in the community divided by all VA‐financed immunotherapy. All costs were measured in inflation‐adjusted 2020 dollars. We examined several secondary measures to explore differences in community‐based immunotherapy service and spending across HOPDs and POs, including receipt of care in the community versus the VA, total number of immunotherapy visits, number of procedures per visit, units per visit, unit costs, the paid amount of the drugs only, and the cost of the drug per visit.

Covariates for the VA compared with community‐based treatment regression included age, sex, race, Medicare enrollment, marital status, the Elixhauser Comorbidity Index, months of treatment, survival at 6 months, rural–urban commuting area codes (RUCA), tertiles of distance to the nearest VA, tertiles of the chemotherapy access measure at the nearest VA facility (defined as the number of chemotherapy treatments, excluding immunotherapy, divided by the number of all patients served at that facility), and VA facility complexity based on the VA Operative Complexity Designation—which classifies medical centers (facilities) by equipment, workload, and staffing.^12^

### 2.3 Statistical analysis

We first examined the proportion of all VA‐financed immunotherapy that was delivered through a community provider as opposed to delivered at a VA site and examined total spending growth of community‐based immunotherapy over this time period. We then estimated a logistic regression model to identify predictors of receiving immunotherapy treatment in the community as opposed to the VA, controlling for the covariates listed above. As a secondary analysis, we compared the means of patient age (*t*‐test), sex (chi‐squared test), treatment costs (Wilcoxon rank‐sum test), treatment duration (Wilcoxon rank‐sum test), and survival of patients (chi‐squared test) who received immunotherapy treatment in the community in HOPDs versus POs for the two most common immunotherapy drugs, Nivolumab and Pembrolizumab.

## 3 RESULTS

**FIGURE 1:**
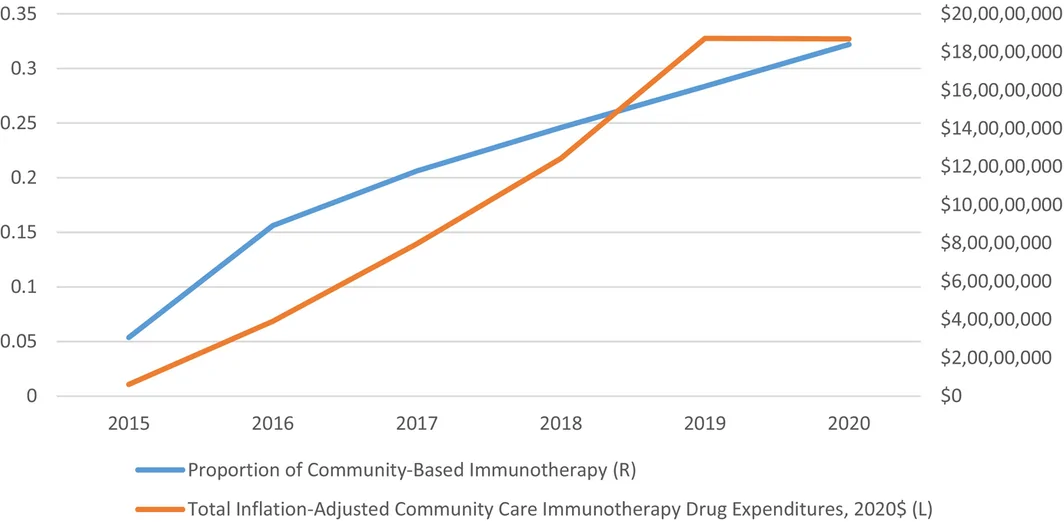
Proportion of all VA‐financed immunotherapy delivered through community care.

Between 2015 and 2020, the proportion of community‐based immunotherapy at the VA increased over sixfold, from 5.3% of all VA‐financed immunotherapy in 2015 to 32.1% in 2020 (Figure 1). During this period, VA annual spending on community‐based immunotherapy grew from $6.1 million to $187 million (2020 $). The most common cancers in our study sample being treated with immunotherapy were respiratory cancers, cancers of the head and neck, gastrointestinal cancers, urinary system cancers, and skin cancers. This was consistent throughout the study period. In unadjusted comparisons, patients who received immunotherapy in the community were more likely to be non‐Hispanic white (*p* < 0.001), be married (*p* < 0.001), reside in a rural area (*p* < 0.001), and to be assigned to a lower complexity VA facility (*p* < 0.001) with a lower chemotherapy access measure (*p* < 0.001) than patients who received immunotherapy in the VA. Black patients were more likely to receive care at the VA (*p* < 0.001). Respiratory cancers were more likely to be treated in the community (*p* < 0.001, while head and neck cancers were more likely to be treated at the VA (*p* < 0.001). Patients who received care in the community had higher mean scores on the Elixhauser Comorbidity Index; a mean of 10.1 among patients who received community‐based immunotherapy compared to 9.6 among those who received VA‐based care (*p* < 0.001) (Table 1).

**TABLE 1:** Characteristics of VA patients who receive immunotherapy in VA facilities versus community‐based care settings, March 2015–November 2020.

Variables	Location of immunotherapy treatment		*p*‐value
	VA	Community	
*N*	15,452	5805	
Age, mean	69.68	70.28	<0.001
Male, %	97.41%	96.45%	<0.001
Medicare enrollee, %	82.32%	84.51%	<0.001
Race			<0.001
White, non‐Hispanic, %	71.67%	78.71%	
Black, %	18.22%	8.96%	
Hispanic, %	3.95%	3.34%	
Asian, Native Hawaiian, and other Pacific Islander, %	0.96%	1.26%	
American Indian and Alaska Native, %	0.74%	1.00%	
Asian, %	0.42%	0.42%	
Race unknown, %	4.19%	5.81%	
Married, %	48.29%	56.09%	<0.001
Respiratory cancer, %	58.39%	62.96%	<0.001
Head and neck cancer, %	45.73%	43.27%	0.001
Gastrointestinal cancer, %	20.84%	19.17%	0.007
Urinary system cancer, %	19.10%	17.83%	0.035
Skin cancer, %	17.38%	18.26%	0.13
Other cancer type, %	33.54%	38.05%	<0.001
Elixhauser Comorbidity Index, mean	9.59	10.11	<0.001
Urban RUCA code, %	66.32%	44.74%	<0.001
Drive distance from nearest VA facility, mean miles	14.84	20.08	<0.001
Facility‐level			
Assigned to complex VA facility, %	49.53%	29.73%	<0.001
Facility chemotherapy access^a^	0.03	0.02	<0.001

^a^ Mean. Defined as # of chemotherapy treatments, excluding immunotherapy, divided by # of enrollees. Calculated at VA sta3n (facility) level.

After adjusting for covariates in a multivariable model, all covariates remained directionally consistent. Respiratory cancers had 19% lower odds of being treated in the VA as opposed to the community, while patients with head and neck cancers had 121% higher odds of being treated in the VA, and urinary system cancers had 118% higher odds of being treated in the VA. Being in the second and third tertiles of the chemotherapy access ratio for chemotherapy was associated with a 239% and 397% increase in the odds of receiving immunotherapy at a VA facility relative to being in the first tertile, respectively (Table 2). Demographic differences between patients who received community‐based versus VA‐based immunotherapy were significant. Male patients had 159% increased odds of being treated at the VA compared to female veterans. Black patients had 181% increased odds of getting treated at the VA as compared to White Non‐Hispanic patients (*p* < 0.001). Being in the third age tertile (as compared to the first tertile) was associated with 17% lower odds of the patient being treated at the VA instead of community care. Married patients had 20% lower odds of being treated at the VA than did single patients. Medicare enrollment was not significantly associated with differences in care setting (Table 2).

**TABLE 2:** Individual and facility‐level predictors of VHA‐based immunotherapy treatment, March 2015–November 2020.

Variables	Odds ratio	*p*‐value	95% CI
Tertile 1: age 19–67	(reference)		
Tertile 2: age 68–73	0.97	0.48	0.88–1.06
Tertile 3: age 74–99	0.83**	0.00	0.75–0.92
Male	1.59**	0.00	1.32–1.92
Medicare enrollee	1.06	0.26	0.96–1.19
White, non‐Hispanic	(reference)		
Black	1.81**	0.00	1.62–2.02
Hispanic	0.87	0.14	0.73–1.04
Asian, Native Hawaiian, and other Pacific Islander	0.85	0.30	0.62–1.16
Asian	0.82	0.38	0.52–1.28
American Indian and Alaska Native	0.86	0.37	0.61–1.20
Race unknown	0.74**	0.00	0.64–0.86
Married	0.80**	0.00	0.75–0.86
Respiratory cancer	0.81**	0.00	0.75–0.88
Head and neck cancer	1.21**	0.00	1.13–1.29
Gastrointestinal cancer	1.03	0.45	0.95–1.13
Urinary system cancer	1.18**	0.00	1.08–1.30
Skin cancer, %	0.97	0.58	0.89–1.07
Other cancer type, %	0.80**	0.00	0.75–0.86
Tertile 1: Elixhauser 1–8	(reference)		
Tertile 2: Elixhauser 9–11	0.84**	0.00	0.77–0.91
Tertile 3: Elixhauser 12–24	0.72**	0.00	0.67–0.78
Urban RUCA code	2.01**	0.00	1.85–2.18
Tertile 1: distance to VA 0–7 miles	(reference)		
Tertile 2: distance to VA 8–18 miles	1.07	0.13	0.98–1.16
Tertile 3: distance to VA, 19–245 miles	0.94	0.22	0.86–1.04
Complex facility	1.66**	0.00	1.55–1.78
Tertile 1: facility chemotherapy access 0–0.2	(reference)		
Tertile 2: facility chemotherapy access 0.02–0.03	2.39**	0.00	2.21–2.58
Tertile 3: facility chemotherapy access 0.3–0.9	3.97**	0.00	3.65–4.32
*N*	21,257		
C‐statistic	0.73		

** *p* < 0.01.

Among patients who received immunotherapy in the community, 41% of patients who received Nivolumab received care in the office setting and 38% of patients who received Pembrolizumab received care in the office setting. Demographic differences in age, sex, and race were similar across office and outpatient hospital settings among individuals receiving either Nivolumab or Pembrolizumab. Respiratory cancer was more likely to be treated in office settings for both drugs (*p* = 0.02 for Nivolumab and *p* = 0.007 for Pembrolizumab), Among skin cancer patients receiving Pembrolizumab, outpatient hospital settings were more common (*p* = 0.002). Approximately one third of the patients died within 6 months of starting treatment; differences in 6‐month mortality across the two settings were not significant among individuals receiving either treatment (Table 3).

**TABLE 3:** Characteristics of patients receiving Nivolumab and Pembrolizumab in office versus outpatient hospital community settings.

	Nivolumab			Pembrolizumab		
	Outpatient Hospital	Office	*p*‐Value	Outpatient Hospital	Office	*p*‐value
*N*	1480	1029		2021	1236	
Age, mean (SD)	68.91 (8.90)	69.97 (8.44)	0.003^A^	70.30 (8.44)	70.71 (8.74)	0.18^A^
Male, *N* (%)	1435 (96.96%)	1004 (97.57%)	0.36^B^	1942 (96.09%)	1195 (96.68%)	0.38^B^
Medicare enrollee (%)	1244 (84.05%)	901 (87.56%)	0.014^B^	1733 (85.75%)	1070 (86.57%)	0.51^B^
Race			<0.001^B^			0.015^B^
Asian, Native Hawaiian, and other Pacific Islander (%)	25 (1.69%)	8 (0.78%)		22 (1.09%)	8 (0.65%)	
Asian, %	10 (0.68%)	2 (0.19%)		18 (0.89%)	4 (0.32%)	
Black (%)	132 (8.92%)	77 (7.48%)		188 (9.30%)	117 (9.47%)	
Hispanic, %	39 (2.64%)	52 (5.05%)		47 (2.33%)	50 (4.05%)	
American Indian and Alaska Native (%)	27 (1.82%)	7 (0.68%)		27 (1.34%)	14 (1.13%)	
Race unknown (%)	116 (7.84%)	61 (5.93%)		133 (6.58%)	99 (8.01%)	
White, non‐Hispanic (%)	1131 (76.42%)	822 (79.88%)		1586 (78.48%)	944 (76.38%)	
Married (%)	828 (55.95%)	577 (56.07%)	0.95^B^	1109 (54.87%)	701 (56.72%)	0.30^B^
Respiratory cancer (%)	772 (52.16%)	585 (56.85%)	0.020^B^	1286 (63.63%)	844 (68.28%)	0.007^B^
Head and neck cancer (%)	557 (37.64%)	410 (39.84%)	0.26^B^	922 (45.62%)	548 (44.34%)	0.47^B^
Gastrointestinal cancer (%)	301 (20.34%)	207 (20.12%)	0.89^B^	385 (19.05%)	211 (17.07%)	0.16^B^
Urinary system cancer (%)	310 (20.95%)	231 (22.45%)	0.37^B^	334 (16.53%)	201 (16.26%)	0.84^B^
Skin cancer (%)	371 (25.07%)	241 (23.42%)	0.34^B^	415 (20.53%)	214 (17.31%)	0.024^B^
Other cancer type (%)	533 (36.01%)	319 (31.00%)	0.009^B^	731 (36.17%)	432 (34.95%)	0.48^B^
Elixhauser Comorbidity Index, mean (SD)	10.17 (3.88)	9.85 (3.82)	0.037^A^	10.14 (3.70)	9.72 (3.66)	0.002^A^
Urban RUCA code (%)	636 (43.0%)	517 (50.2%)	<0.001^B^	840 (41.6%)	623 (50.4%)	<0.001^B^
Months of treatment, mean (SD)	6.50 (7.65)	7.50 (8.70)	0.002^C^	6.27 (6.59)	6.84 (6.63)	0.017^C^
Died within 6 months of starting treatment, *N* (%)	603 (40.7%)	382 (37.1%)	0.068^B^	738 (36.5%)	423 (34.2%)	0.18^B^

*Note*: A = *t*‐test; B = chi Squared test; C = Wilcoxon Rank Sum.

Patients who received community care in HOPDs had significantly higher scores on the Elixhauser Index compared to patients who received care in office settings. On average, treatment durations were slightly longer among patients who received care in office settings for Nivolumab, an average of 7.5 months versus 6.5 months (*p* = 0.005). Differences in treatment duration for Pembrolizumab were 6.3 months among those treated in HOPDs compared to 6.8 among those treated in POs (*p* < 0.001) (Table 3).

Patients who received care in POs had significantly more visits over the course of immunotherapy treatment (Table 4). Patients receiving Nivolumab in POs had on average 11.3 visits compared to 8.5 visits among those who received care in HOPDs (*p* < 0.001). Similarly, for Pembrolizumab, the average number of visits in the office setting was 8.4 compared to 7.2 in HOPD settings (*p* < 0.001). The number of units administered per visit was similar across the two settings, with those in HOPDs receiving slightly higher units than in office settings, for Nivolumab, 313.3 in HOPD versus 294.0 in offices (*p* < 0.001), and for Pembrolizumab, 209.8 in HOPDs versus 211.8 in office settings (*p* = 0.24).

**TABLE 4:** Utilization and costs among patients receiving Nivolumab and Pembrolizumab in office versus outpatient hospital community settings.

	Nivolumab		*p*‐value	Pembrolizumab		*p*‐value
	Outpatient Hospital	Office		Outpatient Hospital	Office	
*N*	1480	1029		2021	1236	
Utilization						
Total visits, mean (SD)	8.52 (10.93)	11.32 (14.14)	<0.001^C^	7.22 (7.67)	8.35 (8.42)	<0.001^C^
Units per visit, mean (SD)	313.32 (150.23)	293.98 (133.48)	<0.001^A^	209.76 (50.55)	211.77 (40.03)	0.24^A^
Total costs, 2020 USD						
Total drug spending per patient during course of treatment, mean (SD)	73163.51 (97780.06)	102569.50 (146960.02)	<0.001^C^	73793.07 (85371.52)	88910.44 (94695.52)	<0.001^C^
Costs per drug unit, 2020 USD						
Unit of drug cost, mean (SD)	27.37 (5.09)	30.72 (17.88)	<0.001^C^	48.45 (9.83)	50.62 (11.25)	<0.001^C^

*Note*: A = *t*‐Test; B = Chi Squared test; C = Wilcoxon rank sum.

Treatment duration was significantly higher in office settings compared to HOPDs for both drugs. The cost per unit of each drug was statistically significantly different but not meaningfully different across the two settings: $27.37 for Nivolumab in HOPDs versus $30.72 in office settings (*p* < 0.001); $48.45 for Pembrolizumab in HOPDs versus $50.62 in office settings (*p* < 0.001). The total drug cost over the course of treatment was higher in office settings than for HOPD settings, due to larger number of visits per patient in office settings ($73163.51 for Nivolumab in HOPDs versus $102569.50 in office settings (*p* < 0.001; $73793.07 for Pembrolizumab in HOPDs versus $88910.44 in office settings (*p* < 0.001) (Table 4).

## 4 DISCUSSION

Immunotherapy has grown as a clinically validated treatment since its first FDA approval in 2011.^13^ Subsequently, more than 1000 clinical trials have been conducted and at least 17 different cancers have been treated.^14^

Community‐based immunotherapy treatment within the VA experienced rapid growth from 2015 to 2020. This mirrors trends globally; worldwide spending on checkpoint inhibitors—the most common type of immunotherapy—grew from $5.6 Billion in 2016 to $35 Billion in 2021.^15^ VA patients who received community‐based immunotherapy were more likely to live farther from a VA facility, reside in a rural area, or live near a VA facility with a lower chemotherapy access measure. This is consistent with prior literature showing that patients who use community care live further from VA facilities and are more likely to live in rural locations than patients who use care at VA sites.^16^

Among VA patients who receive community‐based immunotherapy treatment, drug costs for Nivolumab and Pembrolizumab did not differ widely across HOPDs and POs. This may be due to the fact that the VA pays Medicare rates to community providers. However, patients who received immunotherapy in office settings had more visits than patients who received care in HOPDs, which led to higher overall immunotherapy drug costs over the course of treatment. The HOPD patients in our sample had somewhat higher comorbidity scores than patients who were treated in office settings so it is possible that these sicker patients had lower ability to tolerate immunotherapy side effects and thus needed to withdraw from treatment earlier. This echoes studies based in the private sector which also found that duration of oncology infusion was longer in office settings as compared to hospital outpatient settings.^17^ The similar drug costs between settings contrasts with the private sector where costs are higher in outpatient hospital settings than office settings.^10^

Regarding patient outcomes, some studies suggest that “care fragmentation,” resulting from difficult coordination between community and VA facilities, may lead to poorer health outcomes and higher costs. Care fragmentation may be especially pronounced in specialized cancer treatments (e.g., gynecological care), as there is less robust research on immunotherapy treatment of those conditions.^18^ Our study excluded patients who were treated in multiple settings, so cannot specifically address care fragmentation related to immunotherapy treatment. However, we did find that patients who received community‐based immunotherapy treatment in HOPDs are on average of slightly poorer health status compared to their peers who obtained care in office settings. The cost differences we observed between HOPDs and office settings were driven by longer treatment durations and higher numbers of visits in office settings compared to HOPDs as well as underlying differences in illness severity. This suggests that these drug cost differences were driven by distinct patterns of utilization in the two settings rather than differences in price.

Limitations of this analysis include that it is descriptive in nature; we cannot make determinations about causality. The focus is on drug costs; we were not able to compare facility fees, and do not look at the cost of other oncology care received in combination with the immunotherapy drug. We expect that there may be differences in cost related to care setting that fall outside the scope of this study; these should be examined in future evaluations.

Since we do not have information on cancer staging or prognosis, we are limited in our ability to compare settings with respect to disease severity. Likewise, we are not able to assess the appropriateness of care, or analyze costs outside of the specific immunotherapy drug.

Based on this analysis, preferentially favoring referrals to community care immunotherapy sites based on office or outpatient hospital setting would not have a meaningful impact on drug‐specific spending.^16^, ^19^ Findings in the literature support these analyses, as researchers have yet to find a substantial way to mitigate cost of treatment.^15^ With this in mind, the field is turning toward comparative analyses and rigorous patient screening to mitigate treatment costs.^18^, ^20^, ^21^, ^22^ Nonetheless, given the high cost of oncology treatment, further operational analyses of ways to contain costs without compromising quality of care are warranted.

## AUTHOR CONTRIBUTIONS

**Megan Ellis Price:** Data curation (lead); formal analysis (lead); methodology (supporting); project administration (equal); validation (lead); writing – original draft (lead); writing – review and editing (lead). **Sarah Gordon:** Conceptualization (equal); formal analysis (equal); methodology (equal); supervision (equal); writing – original draft (equal). **Caroline Emmitt:** Investigation (supporting). **Nambi Ndugga:** Conceptualization (supporting); investigation (supporting). **Aigeirm Kabdiyeva:** Data curation (equal). **Hillary Mull:** Methodology (equal); supervision (equal); writing – review and editing (equal). **Steven Pizer:** Conceptualization (lead); methodology (lead); supervision (lead); writing – review and editing (equal). **Melissa M. Garrido:** Conceptualization (lead); methodology (lead); project administration (lead); supervision (lead); writing – review and editing (equal).

## FUNDING INFORMATION

This work was funded by: QUERI PEC 16‐001; HSRD SDR 19‐327.

## CONFLICT OF INTEREST STATEMENT

Sarah Gordon was a senior advisor on health policy in the Office of the Assistant Secretary for Planning and Evaluation at the Department of Health and Human Services. However, this article was conceived and drafted while Dr. Gordon was employed at Boston University, and the findings and views in this article do not reflect the official views or policy of the Department of Health and Human Services.

## Data Availability

The data are not publicly available due to privacy restrictions and Department of Veterans Affairs data use regulations.

## APPENDIX B Sample selection for VHA versus community care analysis.

### B.1

**Table:**

Patient count	Description
24,123	Received immunotherapy between 2015 and 2020
23,928	Remove if missing the driving distance to the closest primary care site
23,919	Remove if the Elixhauser count is null or 0, as we believe this indicates the patient's VA records are incomplete
21,257	Remove if received care for multiple drugs or received immunotherapy in multiple locations on the same day

## APPENDIX C Sample selection for office versus outpatient hospital analysis.

### C.1

**Table:**

Distinct patients	Notes
6108	Observations that are office or outpat hospital Nivo and Pembro between 2015 and 2020
6057	Removed if missing demographic information
5778	Removed if treated for multiple drugs or in multiple locations
5771	Removed if unit cost of drug <1
5766	Removed if unit cost of drug >1000
